# Diverse Metabolites and Pharmacological Effects from the Basidiomycetes *Inonotus hispidus*

**DOI:** 10.3390/antibiotics11081097

**Published:** 2022-08-12

**Authors:** Zhen-xin Wang, Xi-long Feng, Chengwei Liu, Jin-ming Gao, Jianzhao Qi

**Affiliations:** 1 Shaanxi Key Laboratory of Natural Products Chemistry and Biology, College of Chemistry and Pharmacy, Northwest Agriculture and Forestry University, Xiangyang 712100, China; 3346462730@nwafu.edu.cn (Z.-x.W.); elf-nar@nwafu.edu.cn (X.-l.F.); jinminggao@nwafu.edu.cn (J.-m.G.); 2 College of Life Sciences, Northeast Forestry University, Harbin 150040, China; liuchw@nefu.edu.cn

**Keywords:** *Inonotus hispidus*, natural product, biological activity, medicinal fungi

## Abstract

*Inonotus hispidus* mushroom is a popular edible and medicinal mushroom with a long history of use. It is well known as a medicinal fungus with various health benefits for its significant anticancer and immunomodulatory activities. Over the last 60 years, secondary metabolites derived from *I. hispidus* and their biological activities have been discovered and investigated. Structurally, these compounds are mainly polyphenols and triterpenoids, which have anticancer, anti-inflammatory, antioxidant, antimicrobial, and enzyme inhibitor activities. Here, the secondary metabolites derived from *I. hispidus* and their activities were systematically and comprehensively classified and summarized, and the biosynthetic pathway of stylylpyrones was deduced and analyzed further. This review contributes to our understanding of *I. hispidus* and will help with research into natural product chemistry, pharmacology, and the biosynthesis of *I. hispidus* metabolites. According to this review, *I. hispidus* could be a promising source of bioactive compounds for health promotion and the development of functional foods.

## 1. Introduction

**Figure 1:**
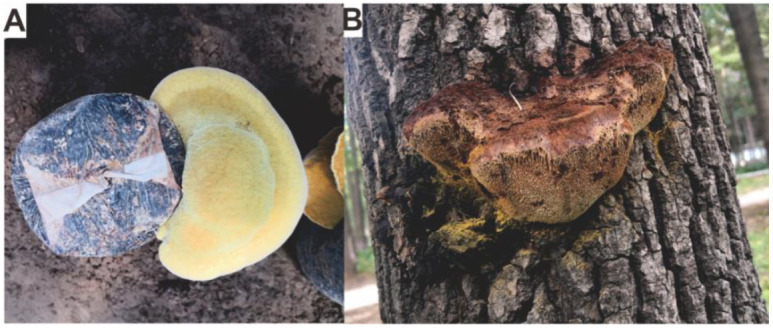
Morphological photographs of fruiting bodies of *I. hispidus* at the initial growth stage in artificial cultivation (**A**) and at the mature stage in the wild (**B**). The photo of mature fruiting body taken on ash on the campus of Northeast Forestry University in August 2021.

The fungus *Inonotus hispidus* (Bull.: Fr.) Karst. is a facultative saprophytic (brown-rot Basidiomycete), which has been found to be parasitic on various broadleaf trees in China and Europe including mulberry (*Morus alba* L.), ash (*Fraxinus mandshurica*), *Populus euphratica*, *Ulmus campestris*, *Sorbus aucuparia,* and *Acer saccharum* [1,2]. According to modern classification systems, *I. hispidus* belongs to the *Inonotus* genus in Hymenochaetaceae (Hymenochaetales, Agaricomycetes, Basidiomycota) [3], whose obsolete synonyms include *Polyporus hispidus* (Bull.) Fr., *Boletus hispidus* (Bull.), *Xanthochrous hispidus* (Bull.) Pat. The fruit bodies of *I. hispidus* are annual, sessile, woody mushroom. The fruiting bodies are bright yellow at the initial growth stage (edible) (Figure 1A), and the color gradually changes from light brown to brown as they grow, becoming dark brown when fully matured with hairy surfaces (Figure 1B), and eventually turns to black [4].

Since *I. hispidus* are frequently parasitic on the mulberry tree (pronounced “sang” in Chinese), their fruit bodies are yellow (pronounced “huang” in Chinese) in color, highly similar in morphology to the modern taxonomic definition of the genus *Sanghuangporus* [5], and relatively close in herbal activity; *I. hispidus* were described as “Sanghuang” in the ancient Chinese *medical classics*. In the Aksu region of Xinjiang and Xiajin County of Shandong in China, *I. hispidus* parasites on mulberry trees has been used as a traditional indigenous medicine “Sanghuang” for the treatment of dyspepsia, cancer, diabetes, and stomach problems [6]. In fact, “Sanghuang” is a traditional collective name including *I. hispidus*, which has a long history of medicinal use and high economic value in the Southeast Asia region (“Meshimakobu” in Japan, and Sanghwang in South Korea) [7]. In China, the earliest medicinal records on “Sanghuang” can be traced back to *Shen Nong’s Materia Medica* in the Qin and Han Dynasties. The “*Shen Nong’s Materia Medica*” and “*Compendium of Materia Medica*” have detailed descriptions of the efficacy of “Sanghuang”. After the Second World War, the number of cancer cases in Nagasaki, Japan, increased dramatically. Danjo-gunto in Nagasaki is rich in “Sanghuang” due to the cultivation of mulberries and silkworms, and the residents were more healthy and had fewer cancer cases due to taking “Sanghuang”. It was later found that it was the anti-tumor properties of mulberry that played a key role [8]. Recent studies in natural product chemistry and pharmacology have shown that *I. hispidus* contains a diverse spectrum of monomeric compounds and exhibits a diversity of physiological actions in in vitro and in vivo assays.

This review summarizes English and Chinese articles published in international academic journals after 1961, based on retrieving electronic databases PubMed, Wiley Online Library, Science Direct, and Web of Science. Totals of 64 compounds, including polyphenols, terpenoids, fatty acids and other types, were classified and sorted. These metabolites with outstanding medicinal value, and crude extracts of the fruit bodies were then inventoried and summarized according to their antitumor, anti-inflammatory, antioxidant, antineurodegenerative, and antimicrobial biological activities. The biosynthetic pathways of styrylpyrones derived from *I. hispidus* and related fungi were deduced and summarized. Related studies on phenols and sesquiterpenes, and food properties of *I. hispidus* are discussed. This work will provide a reference for subsequent studies on the natural product chemistry, pharmacological activities, and biosynthesis of secondary metabolites of *I. hispidus.*

## 2. Compounds with Diverse Structures

### 2.1. Polyphenol Compounds

**Figure 2:**
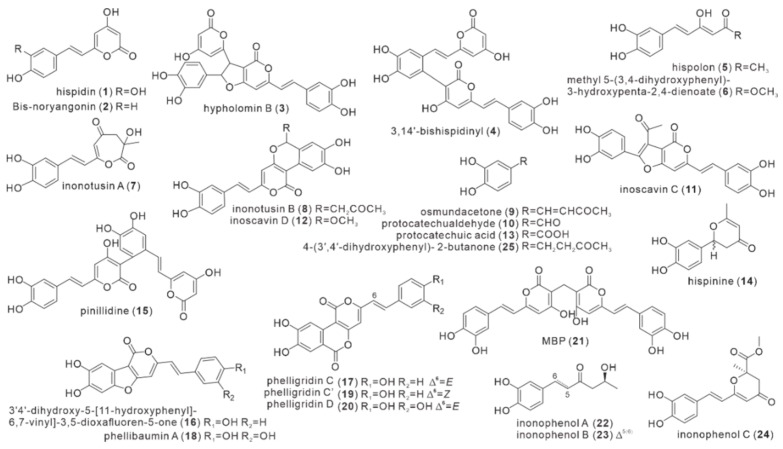
Structures of polyphenol compounds (**1**–**25**).

Polyphenols (Figure 2) are the main pigment components present in the fruiting bodies of *I. hispidus*. Zopf first isolated a series of yellow substances from the fruiting bodies of *I. hispidus* in 1889, and Edwards et al. isolated the yellow substances to obtain a yellow crystalline hispidin (**1**) in 1961 [9]. Hispidin was the first reported monomeric compound from *I. hispidus*, and its structure was identified as 6-(3,4-dihydroxystryl)-4-hydroxy-2-pyrone by infrared techniques and derivatization reactions. Bis-noryangonin (**2**) was found in the fermentation broth of *P. hispidus* (synonym of *I. hispidus*) in liquid medium with glucose as the main carbon source. Its structure was determined as 4-bydroxy-6-(4-hydroxystylyl)-2-pyrone by isotopic label feeding, spectral characterization, and thin-layer chromatography (TLC) [10]. Fiasson and Jean-Louis isolated hispidin (**1**) from the fruiting bodies of *I. hispidus* in 1982, along with its two dimer derivatives, hypholomin B (**3**) and 3,14’-bishispidinyl (**4**) [11]. In 1996, Nasser Ali et al. obtained two pigment polyphenols, hispolon (**5**) and known hispidin (**1**), from the crude ethanolic extract of fruiting bodies under the guidance of immunomodulatory and antiviral activity. The structure of compound **5** was identified as 6-(9,10-dihydroxyphenyl)-3,5(*E*)-hexadien-4-ol-2-one by combined spectroscopic, mass spectrometry (MS) and nuclear magnetic resonance (NMR) techniques [12]. In 2009, Yousfi et al. extracted and isolated two other known polyphenols (**1** and **5**) and a new compound methyl 5-(3,4-dihydroxyphenyl)-3-hydroxypenta-2,4-dienoate (**6**) from the antioxidant components of the fruiting bodies parasitic on *Pistacia atlantica*, and its structure was determined by NMR and EI-MS [13]. In the experiment to determine the trace element composition of anti-influenza virus mushrooms, compounds **1** and **5** were once more isolated and extracted from the fruiting bodies of *I. hispidus* in 2011 [14]. In 2011 and 2012, Zan et al. isolated ten phenolic compounds from methanol extracts of fruiting bodies living on ash, among which inonotusins A (**7**) and B (**8**) were new natural products. The other known compounds were hispidin (**1**), hispolon (**5**), osmundacetone (**9**), protocatechualdehyde (**10**), inoscavin C (**11**), and inoscavin D (**12**), as well as protocatechuic acid (**13**) [15]. Compound **7** contains a highly oxidized functional group with a 2,3,4,5-tetrahydrooxepine backbone, which is the only phenolic compound containing a heptameric ring from *I. hispidus.* A combined approach of MS, spectroscopy and NMR methods clarified the structures of **7** and **8** are 6-(11,12-dihydroxystyryl)-2-hydroxy-2-methyl-5,6-dihydro-oxepine-1,4-dione and 5-(10,11-dihydroxystyryl)-3,4-dihydroxy7-(2-oxopropyl) pyrano-isochromen-1-one [16]. Compounds **9–13** were isolated from this strain for the first time [15]. A content comparison revealed that the fruit bodies contained higher concentrations of hispidin (**1**), hispolon (**5**), and osmundacetone (**9**), which were isolated for the first time from this strain [15]. Gruendemann et al. isolated known compounds hispidin (**1**) and hispolon (**5**) from methanol extracts of their fruiting bodies with the help of bioactivity-guided fractionation and analyzed their immunomodulatory effects in 2016 [17]. In 2017, Ren et al. isolated a new compound hispinine (**14**) and five known polyphenols (**1**, **4**, **5**, **9**, and **15**) from the methanol extract of fruiting bodies of Xinjiang (China) indigenous medicinal fungus *I. hispidus* [18]. Despite the fact that the structure of compound **14** was determined by spectroscopic analysis to be 2-(3,4-dihydroxyphenyl)-6-methyl-2H-pyran-4(3H)-one [18], its absolute conformation has not yet been verified. Compound **15**, pinidine, isolated from *I. hispidus* in 2017, was first isolated from *Phellinus pini* [19]. *Phellinus pini* is a fungus that is morphologically similar to the fungi of the genus *Sanghuangporus* defined by the latest taxonomy [5]. Five polyphenols (**16**–**20**) were isolated and identified by Li et al. from the ethanolic extract of mature yellow fruiting bodies in 2017. Two compounds 3′4′-dihydroxy-5-[11-hydroxyphenyl]-6,7-vinyl]-3,5-dioxafluoren-5-one (**16**) and phelligridin C (**17**) were undescribed previously, while phellibaumin A (**18**), phelligridin C’ (**19**) and phelligridin D (**20**) were isolated for the first time from *I. hispidus* [2]. In 2019, Yang et al. isolated the compound 3,3′-methylene-bis [6-(3,4-dihydroxystyryl)-4-hydroxy-2H-pyran-2-one (MBP, **21**) from a methanol extract of the fruiting body, together with four reported polyphenols (**9, 10**, **13**, and **18**) derived from *I. hispidus* [20]. From a methanol extract of the fruiting body, Kou et al. isolated six polyphenols in 2021, including three compounds inonophenols A-C (**22**–**24**), as well as **5**, **9**, and 4-(3′,4′-dihydroxyphenyl)-2-butanone (**25**) [21]. The structures of compounds **22**–**24** were identified by NMR, HRMS, and/or computational circular dichroism data [21]. In the work on the analysis of antitumor metabolites in the mycelial fermentation broth of *I. hispidus*, hispolon (**5**) was isolated again, which was the first reported isolation from the liquid fermentation [22]. Bis-noryangonin (**2**), isolated from the mycelial broth of *I. hispidus*, is the only phenolic metabolite derived solely from the mycelial broth [10]. Structurally, compound **21**, MBP, is the symmetrical isomer of compound **1**, while compounds **3**, **4**, and **15** are its non-symmetrical isomers.

### 2.2. Triterpenoids

**Figure 3:**
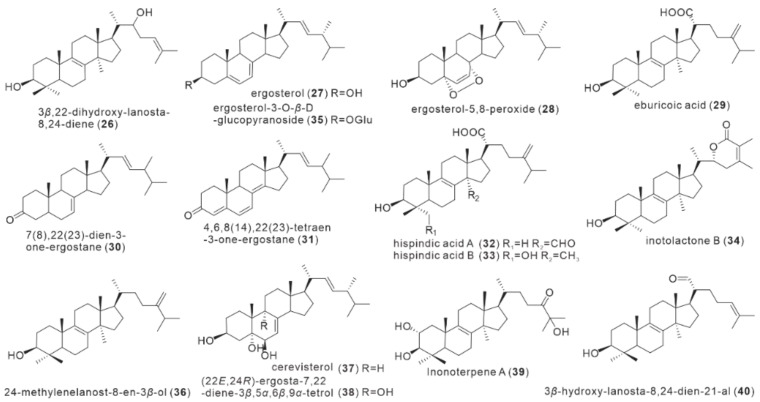
Structures of triterpenoids (**26**–**40**).

Triterpenoids are another important group of secondary metabolites found in *I. hispidus*, with a total of 15 triterpene compounds isolated (Figure 3). Yang et al. first reported the isolation and identification of four triterpenoid monomers from the ethanolic extract of fruiting bodies in 2008, which included 3,22-dihydroxy-lanosta-8,24-diene (**26**), ergosterol (**27**), ergosterol-5,8-peroxide (**28**), and eburicoic acid (**29**), with ergosterol (**27**) being the main chemical constituent isolated from 95% ethanolic extract of *I. hispidus* [23]. Zan et al. isolated five triterpenoids from the methanolic extract of fruiting bodies in 2012, including 7(8),22(23)-dien-3-one-ergostane (**30**), 4,6,8(14),22(23)-tetraen-3-one-ergostane (**31**) for the first time from this fungus, along with known compounds **27–29** [15]. Compound **28** is a high-content component in the fruiting bodies of *I. hispidus* [15].

Ren et al. isolated six triterpenoids, including hispindic acids A and B (**32**–**33**), as well as the known compounds **27**, **29**, inotolactone B (**34**), and ergosterol-3-*O*-*β*-D-glucopyranoside (**35**) from the methanolic extract of fruiting bodies in 2017. The two new triterpenoids **32** and **33** were structurally identified as 24-exomethylene-3*β*-hydroxy-30-oxo-lanost-8-en-21-oic acid and 24-exomethylene-3*β*, 28-dihydroxy-lanost-8-en-21 oic acid by a combination of mass spectrometry and NMR techniques [18]. Seven triterpenoids were isolated from the methanolic extract of the fruiting bodies of *I. hispidus* by Kou et al. in 2021, including compound **28**, **35**, 24-methylenelanost-8-en-3*β*-ol (**36**), cerevisterol (**37**), (22*E*, 24*R*)-ergosta-7,22-diene-3*β*,5*α*,6*β*,9*α*-tetrol (**38**), and Inonoterpene A (**39**), and 3*β*-hydroxy-lanosta-8,24-dien-21-al (**40**). Among them, Inonoterpene A (**39**) is a new Lanostane triterpenoid, and compound **40** was isolated from the fungus for the first time. Interestingly, the above 15 triterpenoids are all lanosterol-type triterpenoids, all derived from the fruiting bodies of *I. hispidus*. Only three triterpenes **32**, **33**, and **39**, have been isolated as new compounds for the first time from *I. hispidus*. Compounds **32** and **40** contain formyl groups at C30 and C21, respectively, while compound **35** possesses glycosylated modifications. It was discovered that specific combinations of four compounds, methyl jasmonate, salicylic acid, oleic acid, and Cu^2+^, and their combinations, can increase the content of total triterpenoids in an orthogonal experiment for liquid fermentation of *I. hispidus* [24].

### 2.3. Fatty Acid Compounds

**Figure 4:**
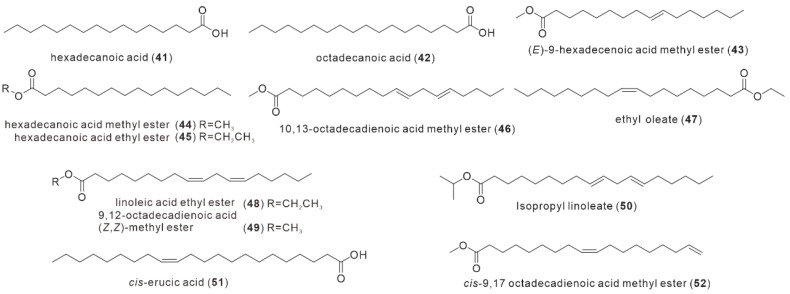
Structures of fatty acid compounds (**41–52**).

The fatty acid analogues of *I. hispidus* were first reported by Yang et al. in 2008, and two monomer compounds hexadecanoic acid (**41**) and octadecanoic acid (**42**) were obtained in the ethanolic extract of its fruit bodies [23] (Figure 4). The structure of the former was identified by EI-MS and TLC comparison of the standard, and the identification of the latter was done with additional infrared techniques [23]. Zan et al. published 2012 the detection results of gas mass spectrometry of the yellow oily substance obtained from the petroleum ether extraction part of the methanol extract of fruit bodies. A total of 12 fatty acid compounds were identified by consulting the Wiley Online Library. These compounds are hexadecanoic acid (**41**), (*E*)-9-hexadecenoic acid methyl ester (**43**), hexadecanoic acid methyl ester (**44**), hexadecanoic acid ethyl ester (**45**), 10,13-octadecadienoic acid methyl ester (**46**), ethyl oleate (**47**), linoleic acid ethyl ester (**48**), 9,12-octadecadienoic acid (*Z*, *Z*)-methyl ester (**49**), Isopropyl linoleate (**50**), *cis*-erucic acid (**51**), and *cis*-9,17 octadecadienoic acid methyl ester (**52**) [15]. Due to their low polarity and insignificant drug properties, fatty acid compounds are not preferred subjects in natural product chemistry studies of fungi. Only two cases of *I. hispidus* have been reported in this regard.

### 2.4. Miscellaneous Compounds

**Figure 5:**
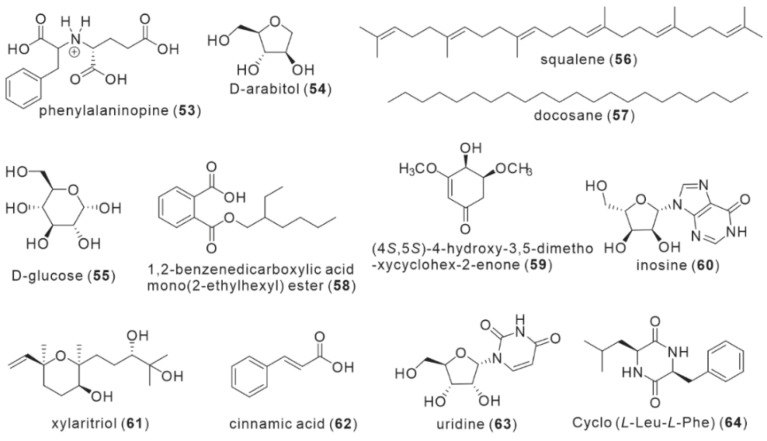
Structures of miscellaneous compounds (**53**–**64**).

During the constituent analysis of dried fruiting bodies of *I. hispidus* in 2007, Politi et al. identified a known compound, phenylalaninopine (**53**), by spectral and mass spectrometric characterization collected by LC-DAD-MS and comparing with the literature, while attempting to develop a phytochemical extraction method for mushrooms using hot water as a solvent [25] (Figure 5). The compound was isolated initially from Clitocybe acromelalga, a poisonous mushroom [26]. Yang et al. obtained two monosaccharides, D-arabitol (**54**) and D-glucose (**55**), along with the isolation of four triterpenoids [23]. In their experiments to examine the fatty acid composition of fruiting bodies, Zan et al. discovered two hydrocarbons, squalene (**56**) and docosane (**57**), and one phthalate, 1,2-benzenedicarboxylic acid mono(2-ethylhexyl) ester (**58**) [15]. In the separation of anticancer active components, Yang et al. isolated a new compound from the methanol extract of fruiting bodies in 2019 and confirmed its structure as (4S,5S)-4-Hydroxy-3,5-dimethoxycyclohex-2-enone (HDE, **59**) by LC-MS and NMR [27]. When analyzing the methanol extract composition of *I. hispidus* fruiting bodies, Yang et al. identified a nucleoside compound, inosine (**60**), based on a comparison of UV absorption characteristics and retention time with a standard [20]. Kou et al. isolated an unsaturated sesquiterpene, xylaritriol (**61**) [21], from the methanol extract of fruiting bodies, which had previously been from the metabolites of endophytic fungus *Xylaria cubensis* (Ascomycete) [28]. Tang et al. isolated compounds, cinnamic acid (**62**), uridine (**63**), and Cyclo (*L*-Leu-*L*-Phe) (**64**) from the fermentation broth of *I. hispidus* mycelium in 2021 [22]. Among these 12 compounds with no shared structural features, compound **59** is the only new compound isolated for the first time from *I. hispidus*, and compound **61** was the only sesquiterpene isolated from *I. hispidus*. Cyclic dipeptide compounds are uncommon in mushrooms (Basidomycota), but compound **64** was discovered in *I. hispidus*. This could be related to the fact that compound **64** is derived from mycelial fermentation broth.

## 3. Biological and Pharmacological Activities

**Figure 6:**
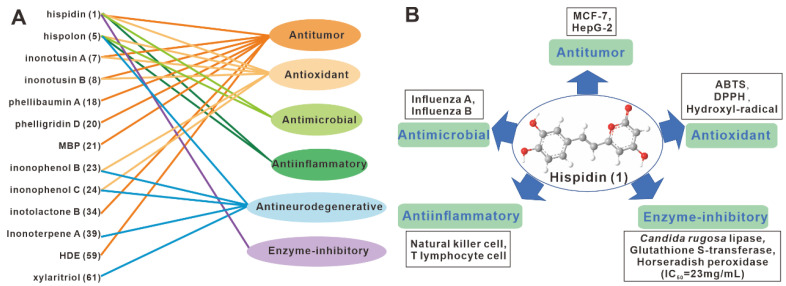
The bioactivities of representative compounds (**A**) and hispidin (**1**) (**B**) from *I. hispidus* compounds.

*Inonotus hispidus* has long been used to treat dyspepsia, cancer, diabetes, and stomach problems in Xinjiang residents [6]. Several studies have been published on the pharmacological activities and mechanisms of major compounds from *I. hispidus* (Table 1). These studies have been summarized here, with a special emphasis on polyphenols with medicinal potential (Figure 6).

### 3.1. Antitumor Activity

As a traditional medicinal fungus with a long-applied history, anti-cancer activity is the principal medicinal value of *I. hispidus*. Multiple monomers with anticancer activity from *I. hispidus* have been discovered and preliminarily resolved in modern chemical chemistry and pharmacological investigations. In vitro screening of five compounds including hispidin (**1**), inonotusin A (**7**) and inonotusin B (**8**) against breast cancer cells MCF-7 using the SRB method revealed that inonotusin A (**7**) had moderate activity against MCF-7 with an IC_50_ value of 19.6 μM [16]. The anti-tumor screening study of MBP (**21**) was carried out by MTT method. The results showed that MBP (**21**) had a dosage-dependent inhibitory effect on the proliferation of HepG2, MCF-7, Hela and A549 cells. Among them, HepG2 showed the best inhibitory effect, with an IC_50_ of 2.3 μg/mL.

**Figure 7:**
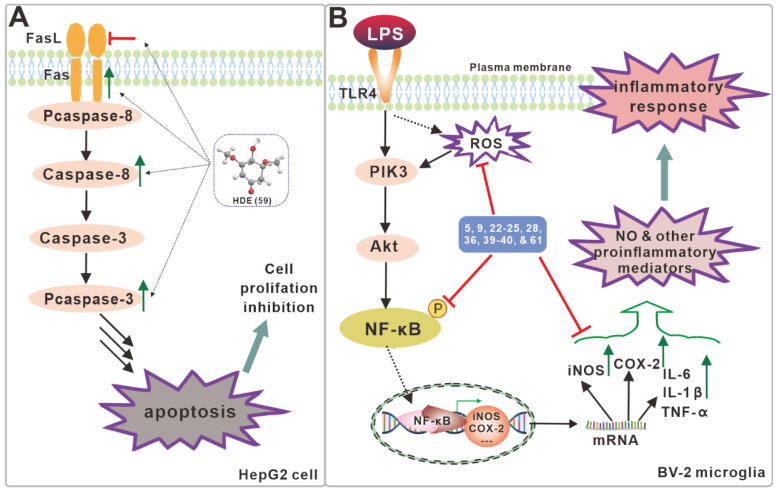
Schematic diagram of the proposed inhibitory mechanism of compound **59** on HepG2 cell (**A**) and compounds including **24** on inflammatory BV-2 microglia induced by LPS (**B**).

According to the results of the apoptosis analysis, it is hypothesized that the compound (**21**) achieves its anti-tumor activity by inducing apoptosis [20]. A study of compound 26’s antitumor activity discovered that it inhibited the growth of lymphoma cells U937, cervical tumor cells Hela, and liver cancer cells QRH-7701 [23]. In vitro screening showed that HDE (**59**) inhibited the proliferation of HepG2, McF-7, Hela, A549 and H_22_ cells, with a greater effect on HepG2 cells (IC_50_ = 7.9μg/mL) [27]. Not only did HDE (**59**) increase the relative activities of Caspase-3 and Caspase-8 and induce HepG2 apoptosis, but it also promoted HepG2 apoptosis by up-regulating *Fas* expression and down-regulating *FasL* expression [27] (Figure 7A). In vivo mouse experiments revealed that HDE (**59**) does not cross the blood–brain barrier and is rapidly metabolized with no adverse effects on organs. Overall, HDE (**59**) has the potential to be an effective antineoplastic agent [27]. At 10 mmol/L, the compound inotolactone B (**34**) still showed activity in activating melanogenesis and tyrosinase in B16 melanoma cells, outperforming 8-MOP (a drug used clinically in the treatment of vitiligo). This indicates that inotolactone B (**34**) has the potential to be developed as an anti-vitiligo agent [18]. The evaluation of the cellular activity of mouse macrophage cells RAW264.7 revealed that compounds **16**, **18** and **20** all had anti-inflammatory activity, with compound **16** being the most active. Cytotoxicity studies showed that all three compounds have a low impact on cell proliferation [2]. This result suggests that compound **16** has the potential to be an anti-leukemic agent. In the supernatant of fermented mycelium of a suspected new subspecies of *I. hispidus*, IH3, a single fraction with high purity of anti-tumor activity, WIH3, was obtained. WIH3 had strong inhibitory against melanoma cells B16 (IC_50_ 29.32 µg/mL), liver cancer cells Hep-3B (37.39 µg/mL), human cervical cancer cells Hela (47.03 µg/mL), and human breast cancer cells MCF-7 (58.01 µg/mL) [33].

In vivo antitumor activity experiments with extracts of fruit bodies from different growth stages of *I. hispidus* revealed that both petroleum ether extracts (IPE) and aqueous extracts at the mature stage had the best tumor suppressive effect on H_22_-bearing mice [15]. Using non-targeted metabolomics techniques, it was found that the antitumor effects of IPE on H_22_ tumor-bearing mice were mainly mediated by energy modulation and regulation of biosynthetic pathways including amino acids and corticosteroids [34]. A series of key regulators of the antitumor activity of IPE were identified in H_22_ tumor suppressor model mice through transcriptomic and proteomic approaches, and the results provide a useful reference for the pharmacological study of the antitumor activity of IPE [35]. The feeding experiment of solid fermentation powder of *I. hispidus* on H_22_-bearing mice demonstrated that it has antitumor effect, whose mechanism may be related to antioxidation, improving immunity and inhibiting tumor tissue angiogenesis [36].

### 3.2. Antioxidant Activity

One of the important properties of mushrooms is their antioxidant function, and antioxidant active molecules derived from *I. hispidus* have been investigated and studied. Antioxidant assays revealed that compounds **1**, **7**, and **8** have significant oxidative protective activity against 2,2′-azino-bis (3-ethylbenzthiazoline)-6-sulfonic acid (ABTS) [16]. Compounds **5**, **9**, **22**–**25** have remarkable inhibitory activity against 1,1-Diphenyl-2-picrylhydrazyl radical (DPPH) with IC_50_ values of 9.82−21.43 μM, with compound **24** being more effective than the positive control [21]. Preliminary structure–activity relationship analysis revealed that catechol unit, double bond on the side-chain, and pyran ring may be important pharmacophores for increasing activity [21]. A study of the antioxidant activity of the compound hispidin (**1**) to scavenge free radicals discovered that at a low concentration (12.5 mg/L), the scavenging rate of **1** to free radical DPPH was 84.70%. At low concentrations, the DPPH scavenging rate was higher than that of BHA, a commercial antioxidant, and compound **1** has natural antioxidant value [30]. The antioxidant activity of compound **1** for scavenging hydroxyl radicals was found to be superior to that of BHA at concentrations as low as 25 mg/kg [30]. DPPH, ABTS and phosphomolybdenum were used as indicators to determine the antioxidant activity of the ethyl acetate extract of *I. hispidus*. The extract was found to be more powerful than the commonly tested antioxidant compounds gallic acid and quercetin [31]. The scavenging effect of hydroxyl radical, DPPH radical and superoxide anion of the volatile oil fragments (H-2) obtained by the diethyl ether reflux extraction of the fruiting bodies of *I. hispidus* was significantly better than that of the volatile oil fragments (H-1) obtained by the steam distillation extraction of the fruiting bodies of *I. hispidus* [37].

### 3.3. Antimicrobial Activity

The antibacterial activity tests performed on hispidin (**1**) revealed that compound **1** lacked adequate inhibitory activity against *Escherichia coli*, *Staphylococcus aureus*, and *Bacillus subtilis* [15]. The inhibition activity of a 70% ethanolic extract of fruit bodies was tested, and it was discovered that the ethanolic extract inhibited only *E. coli*, with the high dosage extract (40.96 mg/mL) achieving a significant level of inhibition. *S. aureus* and *B. subtilis* were not inhibited significantly [15]. At a concentration of 1 × 10^−5^ g/mL, both the H-1 (water extract) and H-2 (ether extract) fractions of fruit bodies inhibited *S. aureus*, *Candida albicans*, *Aspergillus niger*, and *Pseudomonas aeruginosa*, with a positive correlation with concentration, but neither inhibited *E. coli* [15]. The disc diffusion assay of ethanolic extracts of *I. hispidus* fruit bodies revealed a minor antimicrobial effect against *S. aureus*, *E. coli*, and *P. aeruginosa* [38]. Hispolon (**5**) and hispidin (**1**) were discovered to have antiviral activity, inhibiting the growth of influenza A and B viruses [29]. After feeding mice *I. hispidus* mushroom containing compounds **1** and **5**, the anti-influenza virus ability of mice was improved, as was the content of trace elements zinc (194 ± 16.9 mg/kg), selenium, and iron in mouse serum. These metal elements are thought to enhance the antiviral ability of the *I. hispidus* mushroom [14].

### 3.4. Neurotrophic and Neuroprotective Activity

Compounds **5**, **9**, and **22**–**25** exhibited a growth-promoting effect on PC-12 cells. Of these, compounds **23** (33.21 ± 0.8%) and **24** (33.34 ± 1.0%) were the most significant in promoting growth at a concentration of 10 μM [21]. In screening assays for anti-neuroinflammatory activity, compounds **5**, **9**, **22**–**25**, **28**, **36**, **39**–**40**, and **61** all inhibited of LPS-induced NO production in BV-2 microglia, with compound **24** showing significant activity with an IC_50_ value of 11.56 μM. None of the compounds tested toxic [21]. Additional molecular immunological experiments revealed that compounds **5**, **9**, and **22**–**25** inhibited the expression of TLR-4 to down-regulate the NF-*κ*B signaling pathway, and then inhibited the expression of COX-2 and iNOS to reduce inflammation (Figure 7B) [21]. These findings show that these polyphenols and triterpenoids have anti-neurodegenerative activity.

### 3.5. Enzyme Inhibitory Activity

Yousfi et al. discovered in 2013 that the polyphenol-rich ethyl acetate extract of *I. hispidus* fruit bodies was very effective in inhibiting *Candida rugosa* lipase, but the specific inhibitory compounds were unknown [39]. In 2015, they isolated hispidin (**1**) from the fruit bodies and demonstrated that hispidin (**1**) strongly inhibited *C. rugosa* lipase, proposing the use of hispidin (**1**) in the treatment of candidiasis [31]. Hispidin (**1**) was discovered to have anti-peroxidase activity, with an IC_50_ of 23 mg/mL showing a strong competitive inhibition of horseradish peroxidase (HRP) activity. The inhibition mechanism of compound **1** against peroxidases (horseradish and thyroid) was studied using molecular docking simulations in terms of hispidin interaction with amino acid residues, and its drug-forming potential was predicted using ADEMT and Lipinski filtering analyses [40]. According to the combined evaluation, hispidin (**1**) is a more potent irreversible thyroperoxidase inhibitor than the anti-thyroid drug 6-hropylthiouracil [40]. The discovery of this result, to some extent, revealed the mechanism of the antioxidant activity of hispidin (**1**). Furthermore, low doses of ethanolic extracts of *I. hispidus* fruiting bodies increased GST enzyme activity [38]. Compound **34** (IC_50_ = 0.24 mM) has excellent α-glycosidase inhibitory activity and is more potent than the clinically used acarbose (IC_50_ = 0.46 mM) [40]. At the same concentration, the ethyl acetate extract of fruiting bodies had higher α-glucosidase inhibitory activity than the *n*-butanol, methanol, and petroleum ether extracts [41].

### 3.6. Immunomodulatory Effects and Other Biological activities

Cellular activity assays on NK cells showed that compounds **1** and **5** both inhibited the activation and proliferation of activated lymphocytes, but the combination of the two did not enhance this effect [17]. This result demonstrates the immunomodulatory activity of these phenolic compounds. In addition, the fermentation broth exopolysaccharide of *I. hispidus* was shown to attenuate inflammatory responses in mice with acute alcoholic liver disease [42].

## 4. Biosynthetic Progresses on Styrylpyrones

**Figure 8:**
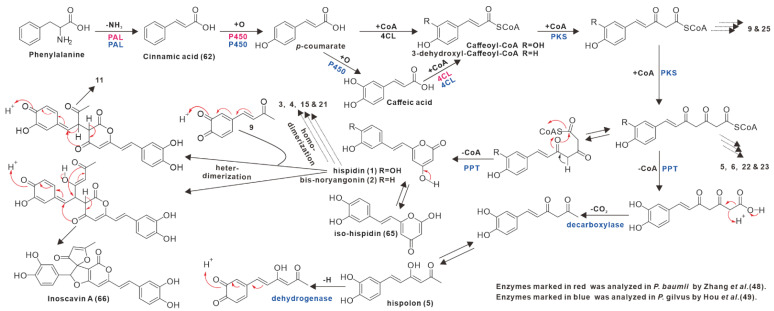
Proposed biosynthesis pathways for styrylpyrones in *Inonotus* and *Phellinus* fungi.

Styrylpyrone compounds are a specific class of polyphenols with a wide range of structures and high yields. The majority of *I. hispidus*-derived yellow polyphenol pigments have a styrylpyrone backbone, and 10 styrylpyrone compounds (**1**, **3**–**4**, **8**, **12**, **15**–**16**, **20**–**21**, **24**) have been identified from the secondary metabolites of *I. hispidus*. Nearly hundreds of styrylpyrone pigments have been discovered in a variety of fungi dominated by the Hymenochaetaceae family since the discovery of hispidin (**1**) as the first naturally occurring styrylpyrone metabolite [43,44,45]. Experiments on light-regulated enzymatic activity revealed that the cinnamic acid (**62**) pathway involved in phenylalanine metabolism is linked to hispidin synthesis in *P. hispidus*. Cinnamic acid (**62**) is thought to be a key intermediate in the biosynthesis of hispidin and other phenylpropanoids [46]. Isotope labeling traces revealed that the styrene unit was incorporated with phenylalanine, tyrosine, cinnamic acid (**62**), p-coumarate, and caffeic acids, while the pyrone ring was incorporated with acetate and malonate [10]. The isolation of five phenylpropanoids from the fruiting bodies of *Inonotus* sp., including iso-hispidin (**65**), inonotic acid methyl ester, and inotilone, provided clues to the biosynthesis of styrylpyrones. The phenylpropanoid polyketide is an important intermediate that is thought to be a side chain elongation of caffeic acid catalyzed by PKS [47]. Integrating metabolomic and proteomic findings that the up-regulation of phenylpropanoid biosynthesis leads to increased levels of hispidin and other polyphenols in *P. baumii*, genes involved in the up-regulation of expression include PAL, C4H, and 4- coumarate-CoA ligase (4CL) (Figure 8) [48]. PAL, 4CL, C4H, polyketide synthase (PKS), palmitoyl protein thioesterase (PPT), decarboxylase, and dehydrogenase genes were identified in a combined investigation of molecular docking simulations and RNA-seq-based analysis of differentially expressed genes in *P. gilvus* (Figure 8) [49]. Based on biosynthetic research and the analysis of existing styrylpyrone compounds derived from the *Phellinus* and *Inonotus* fungi, the comprehensive biosynthetic pathways of styrylpyrone compounds, including hispidin (**1**), hispolon (**5**) and their dimers, as well as inoscavin A (**66**), have been deduced in this review (Figure 8) [47,48,49]. The natural structural diversity of styrylpyrones suggests that their biosynthetic gene clusters are active and contain multiple post-modification genes. It is speculated that the study of its biosynthetic pathway and related synthetic gene clusters may aid in understanding the biosynthetic network of styrylpyrones and their derivatives in *I. hispidus*.

## 5. Discussions and Perspectives

Triterpenes are characteristic pharmacological components of lignified traditional medicinal fungi, such as Ganoderic acid from *Ganoderma* species [50], and Eburicoic acid and its derivatives from polypore species [51]; however, they are not monolithic. Phenols, a class of high-value natural products with medicinal potential [44,52], are prominent bioactive components of the highly lignified and well-known medicinal mushrooms of the genus *Phellinus* and *Inonotus* belonging to the order of Hymenochaetales [43]. The diversely modified phenolic derivatives yielded by these mushrooms have promising applications in drug discovery and development [43,44]. Hispidin (**1**) is the first compound isolated and identified from *I. hispidus*, and these phenolic compounds with styryl and pyrone moieties are gathered here with diverse activities such as antitumor, antioxidant, antimicrobial, and anti-inflammatory activities (Figure 6). These diverse activities of the natural product reflect the biological activities of its producer. Despite the fact that **5** was initially isolated from *I. hispidus* in 1996 [12], it is more of a characteristic component of fungi belonging to the genus *Phellinus*, as it has also been isolated from various species of the genus *Phellinus*, including *P. linteus*, *P. igniarius*, *P. lonicerinus* and *P. merrillii* [43]. A summary of the anticancer, antidiabetic, antioxidant, antiviral, and anti-inflammatory properties of compound **5** revealed its potential development value as a complementary and alternative medicine [43].

As one of the most abundant secondary metabolites found in mushrooms, numerous mushroom-derived sesquiterpenes have been isolated and identified [53]. Sesquiterpene synthases are one of the core gene types involved in the biosynthesis of secondary metabolites. Mushroom genomes contain typically at least ten sesquiterpene synthases. *I. obliquus* is a congener species of *I. hispidus.* Although the genome of *I. obliquus* contains at least 4 types of 20 sesquiterpene synthases [54], only 8 sesquiterpenoids of the drimane-type have been reported [55]. So far, only one sesquiterpenoid (**61**) has been reported for *I. obliquus* [21], and the type and number of sesquiterpene synthases are also unknown. The identification of sesquiterpene cyclases derived from lots of mushrooms including *Coprinus cinereus* [56], *Omphalotus olearius* [57], *Stereum hirsutum* [58,59], *Flammulina velutipes* [60], and *Agrocybe aegerita* [61] have led to the discovery of numerous novel sesquiterpenoids, which are not found in their natural producer instead. As a result, we speculate that in most mushrooms, only one or a few types of sesquiterpene synthases can be expressed to produce specific sesquiterpenoids, while the majority of sesquiterpene synthases are silent and have no corresponding products.

Although *I. hispidus* has been widely considered a traditional medicinal fungus due to its secondary metabolites with remarkable significant pharmacological value, the food properties of *I. hispidus* that are rich in health and nutritional functional factors should not be overlooked. The edible fruiting body of *I. hispidus* is a scarce ingredient. *I. hispidus* possess a good selenium-enriching function [62], and the antiviral ability of *I. hispidus* rich in selenium has also been enhanced. The dihydroxy phenylalanine-melanin contained in *I. hispidus* could effectively remove DPPH radicals and can therefore be used in health food or as a food additive [63]. Furthermore, the extracellular exopolysaccharide of *I. hispidus* was investigated and found to have antioxidant activity [64] and hepatoprotective functions [42].

Mushrooms are often perceived more for their anticancer or immunomodulatory activity than for their antibacterial activity. In fact, mushrooms with antimicrobial activity are uncommon [65,66], with *Pleurotus mutilus* and *I. hispidus* being the few examples. Pleuromutilin was found to have good antifungal inhibitory activity and was used as the lead compound in the development of the human antibiotic retapamulin, which was approved by the FDA in 2007 [67]. Both aqueous and ether extracts of *I. hispidus* mushrooms inhibit pathogenic microorganisms such as *S. aureus* [15]; however, monomeric compounds with antibacterial activity have yet to be identified. As a result, the discovery and development of antimicrobial compounds derived from *I. hispidus* is worth pursuing.

Herein, a total of 64 compounds derived from *I. hispidus* were collected and classified according to their structural features. Biological and pharmacological activities including antitumor, antioxidant, as well as anti-inflammatory and neurotrophic and neuroprotective activities were summarized. These studies, however, were limited to the isolation, identification, and activity evaluation of compounds. Current research has not paid enough attention to *I. hispidus*, despite its significant medicinal efficacy and functional food value. Although the genomes of multiple rare medicinal fungi including *I. obliquus* [54] have been sequenced, the genome of *I. hispidus* has not been sequenced. The lack of genomic information of *I. hispidus* severely hampered the biosynthetic pathway elucidation of its active compounds. As a result, it is critical to complete the genome sequencing of *I. hispidus* [54] using rapidly developing sequencing technology, which will lay a firm foundation for biosynthesis research and synthetic biology production of valuable compounds to meet the needs of the drug development and functional food industries.

## Figures and Tables

**Table 1:** Occurrence of compounds from *Inonotus hispidus*.

No.	Name	Resource	Method	Activity	Reference
**1**	hispidin	fruiting body, mycelium	EtOH, MeOH	Antitumor, ABTS, DPPH, influenza A and B viruses, Enzyme-inhibitory, antineurodegenerative	Edwards et al. [9]. Zan et al. [15]. Ali, N.A.A. et al. [29]. Zan et al. [16]. Zan et al. [30]. Benarous, K. et al. [31]. Benarous, K. et al. [32]. Gruendemann, C. et al. [17].
**2**	Bis-noryangonin	mycelium	NA	NA	Perrin, P.W.et al. [10].
**3**	hypholomin B	fruiting body	NA	NA	Fiasson et al. [11].
**4**	3,14’-bishispidinyl	fruiting body	MeOH	NA	Fiasson et al. [11]. Ren et al. [18].
**5**	hispolon	fruiting body mycelium	EtOH, MeOH EAC	DPPH, influenza A and B viruses, PC-12 cell, BV-2 microglia, ntineurodegenerative	Nasser Ali et al. [12]. Zan et al. [15]. Tang et al. [22]. Kou et al. [21]. Ali, N.A.A. et al. [29]. Gruendemann, C. et al. [17].
**6**	methyl 5-(3,4-dihydroxyphenyl)-3-hydroxypenta-2,4-dienoate	fruiting body	MeOH	NA	Yousfi et al. [13].
**7**	inonotusin A	fruiting body	MeOH	MCF-7, ABTS	Zan et al. [15]. Zan et al. [16].
**8**	inonotusin B	fruiting body	MeOH	Antitumor, ABTS	Zan et al. [15]. Zan et al. [16].
**9**	osmundacetone	fruiting body	MeOH	DPPH, PC-12 cell, BV-2 microglia	Zan et al. [15]. Kou et al. [21].
**10**	protocatechualdehyde	fruiting body	MeOH	NA	Zan et al. [15].
**11**	inoscavin C	fruiting body	MeOH	NA	Zan et al. [15].
**12**	inoscavin D	fruiting body	MeOH	NA	Zan et al. [15].
**13**	protocatechuic acid	fruiting body	MeOH	NA	Zan et al. [15].
**14**	hispinine	fruiting body	MeOH	NA	Ren et al. [18].
**15**	pinillidine	fruiting body	MeOH	NA	Ren et al. [18].
**16**	3’4’-dihydroxy-5-[11-hydroxyphenyl]-6,7-vinyl]-3,5-dioxafluoren-5-one	fruiting body	EtOH	mouse macrophage cell	Li et al. [2].
**17**	phelligridin C	fruiting body	EtOH	NA	Li et al. [2].
**18**	phellibaumin A	fruiting body	EtOH MeOH	mouse macrophage cell	Li et al. [2]. Yang et al. [20].
**19**	phelligridin C’	fruiting body	EtOH	NA	Li et al. [2].
**20**	phelligridin D	fruiting body	EtOH	mouse macrophage cell	Li et al. [2].
**21**	MBP	fruiting body	MeOH	HepG2, MCF-7, Hela and A549 cells	Yang et al. [20].
**22**	inonophenol A	fruiting body	MeOH	DPPH, PC-12 cell, BV-2 microglia	Kou et al. [21].
**23**	inonophenol B	fruiting body	MeOH	DPPH, PC-12 cell, BV-2 microglia	Kou et al. [21].
**24**	inonophenol C	fruiting body	MeOH	DPPH, PC-12 cell, BV-2 microglia	Kou et al. [21].
**25**	4-(3′,4′-dihydroxyphenyl)-2-butanone	fruiting body	MeOH	DPPH, PC-12 cell, BV-2 microglia	Kou et al. [21].
**26**	3*β*, 22-dihydroxy-lanosta-8,24-diene	fruiting body	EtOH	U937, Hela, QRH-7701	Yang et al. [23].
**27**	ergosterol	fruiting body mycelium	EtOH, MeOH EAC	NA	Yang et al. [23]. Zan et al. [15]. Tang et al. [22].
**28**	ergosterol-5,8-peroxide	fruiting body	EtOH, MeOH	BV-2 microglia	Yang et al. [23]. Zan et al. [15]. Kou et al. [21].
**29**	eburicoic acid	fruiting body	EtOH, MeOH	NA	Yang et al. [23]. Zan et al. [15].
**30**	7(8),22(23)-dien-3-one-ergostane	fruiting body	MeOH	NA	Zan et al. [15].
**31**	4,6,8(14),22(23)-tetraen-3-one-ergostane	fruiting body	MeOH	NA	Zan et al. [15].
**32**	hispindic acid A	fruiting body	MeOH	NA	Ren et al. [18].
**33**	hispindic acid B	fruiting body	MeOH	NA	Ren et al. [18].
**34**	inotolactone B	fruiting body	MeOH	B16 melanoma cell	Ren et al. [18].
**35**	ergosterol-3-*O*-*β*-D-glucopyranoside	fruiting body	MeOH	BV-2 microglia	Ren et al. [18]. Kou et al. [21].
**36**	24-methylenelanost-8-en-3*β*-ol	fruiting body	MeOH	BV-2 microglia	Kou et al. [21].
**37**	cerevisterol	fruiting body	MeOH	NA	Kou et al. [21].
**38**	(22*E*, 24*R*)-ergosta-7,22-diene-3*β*,5*α*,6*β*,9*α*-tetrol	fruiting body	MeOH	NA	Kou et al. [21].
**39**	Inonoterpene A	fruiting body	MeOH	BV-2 microglia	Kou et al. [21].
**40**	3*β*-hydroxy-lanosta-8,24-dien-21-al	fruiting body	MeOH	BV-2 microglia	Kou et al. [21].
**41**	hexadecanoic acid	fruiting body	EtOH, MeOH	NA	Yang et al. [23]. Zan et al. [15].
**42**	octadecanoic acid	fruiting body	EtOH	NA	Yang et al. [23].
**43**	(*E*)-9-hexadecenoic acid methyl ester	fruiting body	MeOH	NA	Zan et al. [15].
**44**	hexadecanoic acid methyl ester	fruiting body	MeOH	NA	Zan et al. [15].
**45**	hexadecanoic acid ethyl ester	fruiting body	MeOH	NA	Zan et al. [15].
**46**	10,13-octadecadienoic acid methyl ester	fruiting body	MeOH	NA	Zan et al. [15].
**47**	ethyl oleate	fruiting body	MeOH	NA	Zan et al. [15].
**48**	linoleic acid ethyl ester	fruiting body	MeOH	NA	Zan et al. [15].
**49**	9,12-octadecadienoic acid (*Z*, *Z*)-methyl ester	fruiting body	MeOH	NA	Zan et al. [15].
**50**	Isopropyl linoleate	fruiting body	MeOH	NA	Zan et al. [15].
**51**	*cis*-erucic acid	fruiting body	MeOH	NA	Zan et al. [15].
**52**	*cis*-9,17 octadecadienoic acid methyl ester	fruiting body	MeOH	NA	Zan et al. [15].
**53**	phenylalaninopine	fruiting body	Water	NA	Politi et al. [25].
**54**	D-arabitol	fruiting body	EtOH	NA	Yang et al. [23].
**55**	D-glucose	fruiting body	EtOH	NA	Yang et al. [23].
**56**	squalene	fruiting body	MeOH	NA	Zan et al. [15].
**57**	docosane	fruiting body	MeOH	NA	Zan et al. [15].
**58**	1,2-benzenedicarboxylic acid mono(2-ethylhexyl) ester	fruiting body	MeOH	NA	Zan et al. [15].
**59**	HDE	fruiting body	MeOH	HepG2, McF-7, Hela, A549 and H22 cells	Yang et al. [27].
**60**	inosine	fruiting body	MeOH	NA	Yang et al. [20].
**61**	xylaritriol	fruiting body	MeOH	BV-2 microglia	Kou et al. [21].
**62**	cinnamic acid	mycelium	NA	NA	Tang et al. [22].
**63**	uridine	mycelium	NA	NA	Tang et al. [22].
**64**	Cyclo (*L*-Leu-*L*-Phe)	mycelium	NA	NA	Tang et al. [22].

NA indicated the data is not available.
