# Correction: Wang et al. Diverse Metabolites and Pharmacological Effects from the Basidiomycetes *Inonotus hispidus*. *Antibiotics* 2022, *11*, 1097

**DOI:** 10.3390/antibiotics11111671

**Published:** 2022-11-21

**Authors:** Zhen-xin Wang, Xi-long Feng, Chengwei Liu, Jin-ming Gao, Jianzhao Qi

**Affiliations:** 1 Shaanxi Key Laboratory of Natural Products and Chemical Biology, College of Chemistry and Pharmacy, Northwest Agriculture and Forestry University, Yangling 712100, China; 2 College of Life Sciences, Northeast Forestry University, Harbin 150040, China

## Error in Authors’ Names and Affiliation

In the original publication, there were publisher errors in the names of authors Zhen-xin Wang, Xi-long Feng and Jin-ming Gao and affiliation (1) of the first, second, fourth and fifth authors (name of the Key Laboratory and city). This correction was approved by the Academic Editor. The original publication has also been updated.

## Error in Figure

In the original publication [1], there was a mistake in **Figure 2** as published. The correct structure of compound **2** was not listed, and structures **2**–**24** did not match the corresponding descriptions in the article. The corrected **Figure 2** appears below.

In the original publication, there was a mistake in **Figure 8** as published. The text notes in the lower right corner of this figure should cite references 48 and 49, not 47 and 48, respectively. The corrected **Figure 8** appears below. 

The authors state that the scientific conclusions are unaffected. This correction was approved by the Academic Editor. The original publication has also been updated.

## Figures and Tables

**Figure 2:**
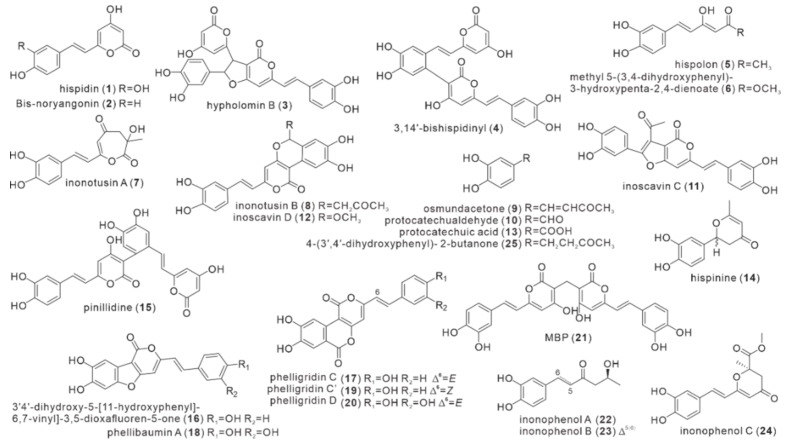
Structures of polyphenol compounds (**1**–**25**).

**Figure 8:**
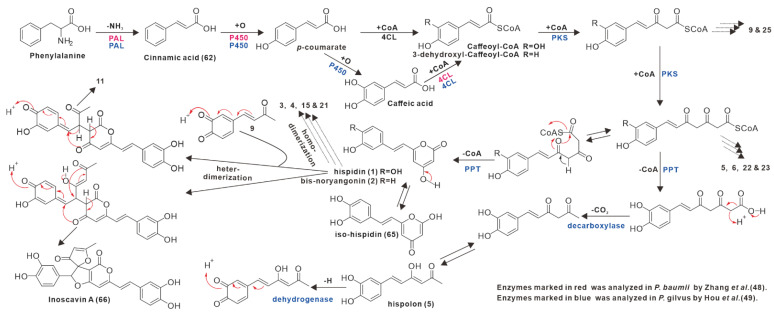
Proposed biosynthesis pathways for styrylpyrones in *Inonotus* and *Phellinus* fungi.
